# Targeting chaperone modifications: Innovative approaches to cancer treatment

**DOI:** 10.1016/j.jbc.2024.107907

**Published:** 2024-10-19

**Authors:** Mariah Stewart, Jonathan C. Schisler

**Affiliations:** 1 The McAllister Heart Institute and Department of Pharmacology, The University of North Carolina at Chapel Hill, Chapel Hill, North Carolina, USA; 2 The Department of Pathology and Lab Medicine and Computational Medicine Program, The University of North Carolina at Chapel Hill, Chapel Hill, North Carolina, USA

**Keywords:** heat shock proteins, cancer, posttranslational modifications, co-chaperones, protein quality control

## Abstract

Cancer and other chronic diseases are marked by alterations in the protein quality control system, affecting the posttranslational destiny of various proteins that regulate, structure, and catalyze cellular processes. Cellular chaperones, also known as heat shock proteins (HSPs), are pivotal in this system, performing protein triage that often determines the fate of proteins they bind to. Grasping the regulatory mechanisms of HSPs and their associated cofactors is crucial for understanding protein quality control in both healthy and diseased states. Recent research has shed light on the interactions within the protein quality control system and how post-translational modification govern protein interactions, function, and localization, which can drive or inhibit cell proliferation. This body of work encompasses critical elements of the heat shock response, including heat shock protein 70, heat shock protein 90, carboxyl-terminus of HSC70 interacting protein, and heat shock protein organizing protein. This review aims to synthesize these advancements, offering a holistic understanding of the system and its response when commandeered by diseases like cancer. We focus on the mechanistic shift in co-chaperone engagement—transitioning from heat shock protein organizing protein to carboxyl-terminus of HSC70 interacting protein in association with heat shock protein 70 and heat shock protein 90—which could influence cellular growth and survival pathways. A comprehensive examination of posttranslational modification–driven regulation within the protein quality control network is presented, highlighting the roles of activation factors, chaperones, and co-chaperones. Our insights aim to inform new strategies for therapeutically targeting diseases by considering the entire heat shock response system.

## Protein quality control and the heat shock response system

**Figure 1:**
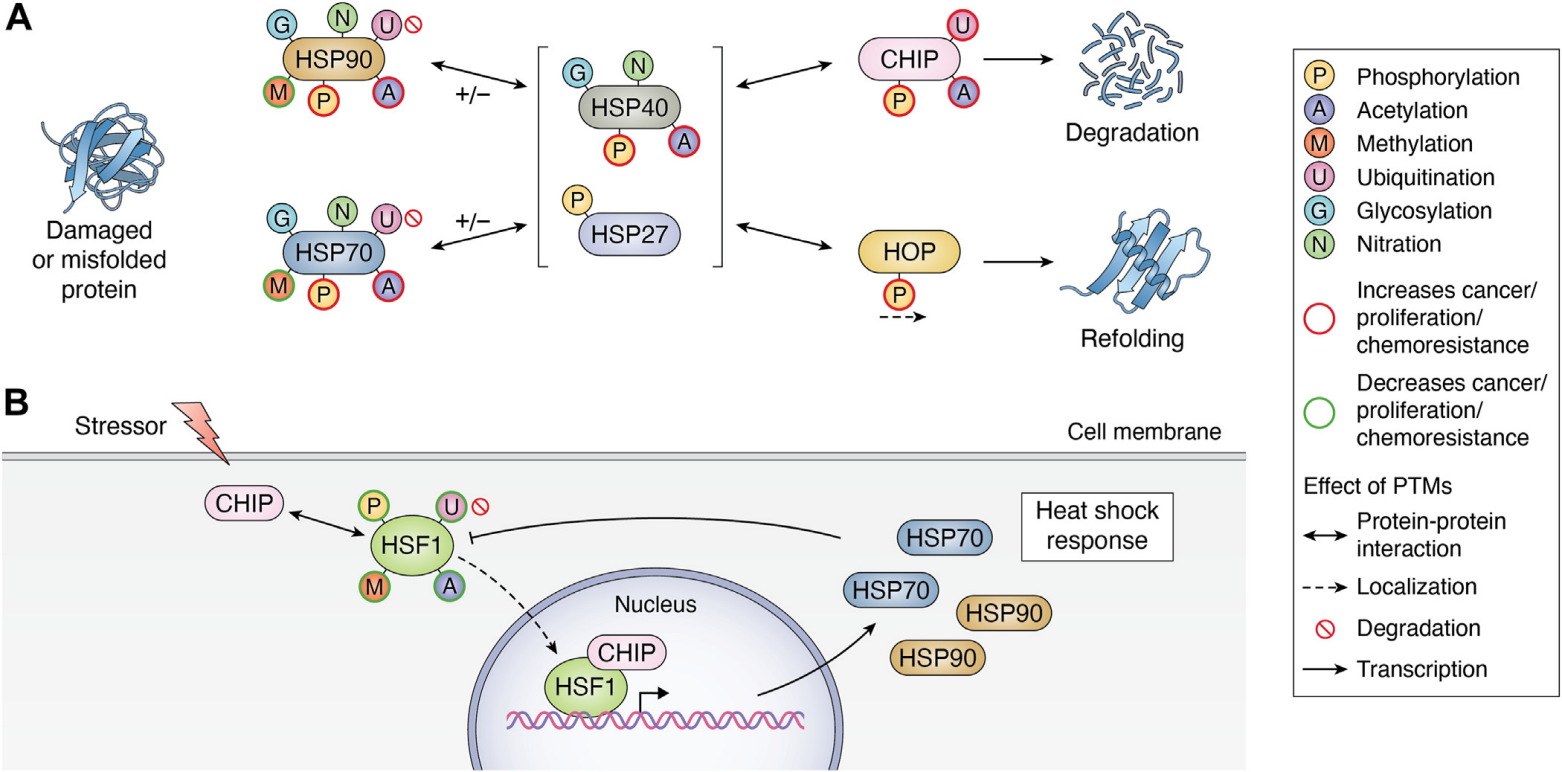
**Mechanisms of the heat shock response and its implications in cancer.***A*, damaged or misfolded proteins are triaged by HSP70 or HSP90, with or without co-chaperones HSP40 or HSP27. The preference for CHIP *versus* HOP determines the fate of substrates, leading to either degradation or refolding. *B*, a stressor promotes the interaction of CHIP with HSF1 to initiate the HSR, leading to HSF1’s translocation to the nucleus. HSF1 then activates the transcription of HSR components. Posttranslational modifications (PTMs) on HSR components can influence protein–protein interactions, localization, degradation, and transcription. These PTMs can also affect cancer proliferation and chemoresistance.

The cellular protein quality control (PQC) system is a complex network of enzymes and organelles crucial for maintaining cellular functions and responding to various stressors (1, 2). Within the PQC system, the heat shock response system (HSR) is critical in managing cellular stress, including heat, oxidative, metabolic, and hypoxia (3). The HSR operates through a sophisticated interplay of molecular chaperones, co-chaperones, transcription factors, and co-factors to ensure protein homeostasis and effectively respond to stressors (4). Central to the HSR are molecular chaperones like heat shock protein 70 (HSP70) and heat shock protein 90 (HSP90), which work in concert with co-chaperones to triage proteins for refolding or degradation (Fig. 1*A*) (5, 6, 7).

The HSR is finely regulated by transcription factors such as heat shock factor 1 (HSF1), which orchestrates the transcriptional response to stress by upregulating various chaperones (8, 9). Upon stress induction, HSF1 changes protein–protein interactions and post-translational modifications (PTMs), leading to its activation and subsequent binding to DNA to promote the transcription of HSR proteins (Fig. 1*B*) (10). Notably, a negative feedback loop involving the chaperone HSP70 and co-chaperone heat shock protein 40 (HSP40, or J-domain proteins) regulates HSF1 activity, preventing excessive transcription of HSR genes (Fig. 1*B*) (11). Furthermore, PTMs play a significant role in modulating the HSR, with studies highlighting the impact of PTMs such as phosphorylation, acetylation, methylation, ubiquitination, glycosylation, and nitration on the system (Fig. 1) (12).

The HSR system operates through two primary chaperone complexes, anchored by HSP70 and HSP90, each utilizing a network of co-chaperones that influence whether proteins are directed toward refolding or degradation pathways (Fig. 1*A*) (13). In cases where degradation is necessary, the ubiquitin-proteasome system is the primary mechanism for disposing of damaged or misfolded proteins marked by ubiquitin (14, 15). The HSR system can also utilize chaperone-mediated autophagy and the lysosomal pathway to degrade larger aggregated substrates, further contributing to cellular proteostasis (14, 16). The balance between refolding and degradation of chaperone-bound substrates is critical for maintaining cellular PQC and overall cellular health (17).

The HSR is not confined to responding solely to heat stress but is also activated by various other stressors like infection or oxidants, showcasing its adaptability in combating different types of cellular stress (18). The intricate regulation of the HSR involves a network of molecular interactions and signaling cascades that ensure a timely and appropriate response to stress conditions (19, 20). Moreover, the involvement of co-chaperones like the carboxyl-terminus of HSP interacting protein (CHIP) and HSP organizing protein (HOP) in fine-tuning protein triage underscores the complexity of the HSR system and its role in maintaining cellular proteostasis (Fig. 1) (12, 21, 22, 23).

The HSR system represents a sophisticated cellular defense mechanism pivotal in managing PQC and responding to diverse stressors. Through the coordinated actions of molecular chaperones, co-chaperones, transcription factors, and PTMs, the HSR ensures the maintenance of proteostasis and cellular health in challenging environmental conditions. Understanding the intricate mechanisms underlying the HSR sheds light on fundamental cellular processes and holds promising implications for therapeutic interventions targeting PQC pathways in various diseases.

While previous reviews have provided valuable insights into the PTMs of individual HSR components (7, 8, 24, 25), our review aims to present a comprehensive mechanistic model that integrates these modifications within a broader system. Focusing on the pivotal molecular switch involving HSP70 and HSP90 with CHIP and HOP, we highlight the nuanced regulation of protein triage and the diverse PTMs that influence the HSR. Additionally, we explore the functional implications of HSR activity on cancer cell dynamics and discuss recent pharmacological advancements targeting HSR components and specific PTMs. This review identifies critical knowledge gaps (***bold and italicized text***) and emphasizes balancing CHIP- and HOP-containing chaperone complexes (underlined text).

### Heat shock protein 70

**Figure 2:**
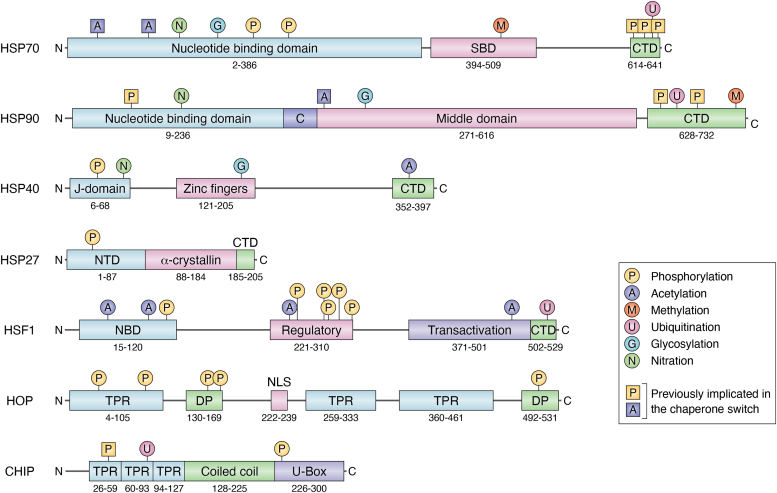
**Components of the heat shock response and posttranslational modifications.** The figure illustrates various domains involved in the HSR, including the substrate-binding domain (SBD), C-terminal domain (CTD), charged domain (C), N-terminal domain (NTD), nucleotide-binding domain (NBD), tetratricopeptide repeat domains (TPR), Asp-Pro rich domain (DP), and nuclear localization sequence (NLS). Posttranslational modifications (PTMs) that impact the co-chaperone switch are indicated with *squares*. The domain boundaries for each protein are specified according to the following UniProt accessions: HSP70 (P11142 · HSP7C_HUMAN), HSP90 (P07900 · HS90A_HUMAN), HSP40 (P31689 · DNJA1_HUMAN), HSP27 (P04792 · HSPB1_HUMAN), HSF1 (Q00613 · HSF1_HUMAN), HOP (P31948 · STIP1_HUMAN), and CHIP (Q9UNE7 · CHIP_HUMAN).

HSP70 is a crucial chaperone protein that plays a significant role in PQC within the cell. Structurally, HSP70 consists of a nucleotide-binding domain, a substrate-binding domain, and a C-terminal domain (CTD) that harbors essential regulatory elements like the lid domain and the EEVD motif for co-chaperone interactions (Fig. 2) (6). One of the primary functions of HSP70 is to recognize misfolded proteins by binding to exposed hydrophobic amino acid residues (4). Refolding of the substrate is catalyzed by repeated cycles of HSP70-substrate binding and release in an ATP-dependent manner (26). The energy released during ATP hydrolysis facilitates the refolding of the substrate, with the subsequent hydrolysis of ATP to ADP stabilizing the protein in place (27).

Moreover, the hydrolysis of ATP by HSP70 is a relatively slow process, necessitating the involvement of co-chaperones like HOP and J-domain proteins to stimulate ATP hydrolysis by stabilizing the HSP70–substrate complex (10). Nucleotide exchange factors further promote nucleotide exchange, releasing both ADP and the substrate for subsequent cellular processes (27). This intricate mechanism highlights the dynamic nature of HSP70 in managing protein folding and quality control within the cell.

### Heat shock protein 90

HSP90 represents another essential component of the HSR system, working alongside HSP70 to ensure proper protein folding and cellular homeostasis (28). HSP90 shares some primary structural similarities with HSP70, featuring a nucleotide-binding domain, a middle domain, and a CTD with an EEVD motif (Fig. 2) (29, 30). Like HSP70, HSP90 also binds ATP, albeit through different structural classes of binding sites, with HSP70 having a hexokinase fold and HSP90 a gyrase fold (31, 32).

One of HSP90's essential functions is stabilizing and activating a specific set of client proteins, thereby influencing various cellular processes (16, 24, 33). HSP90 interacts with co-chaperones to determine the fate of substrates, whether they undergo degradation, refolding, or modification, ultimately contributing to either pro-degradation or pro-folding cellular environments (34). Interestingly, HSP90 can complement the actions of HSP70 by participating in a secondary stage of the refolding process, enhancing protein folding and aiding in the final steps of protein maturation (33, 35).

### HSP70 and HSP90, ying and yang

While HSP70 and HSP90 are critical players in the cellular PQC system, they exhibit distinct tertiary structural features and functional roles within the cell (19). HSP70 primarily recognizes and binds to misfolded proteins, initiating the refolding process through ATP-dependent mechanisms (27). In contrast, HSP90 stabilizes and activates specific client proteins, collaborating with co-chaperones to modulate substrate fate (36). Despite these functional differences, both chaperones share commonalities in their ATP-binding capabilities, highlighting the importance of nucleotide-dependent processes in protein folding and quality control (37).

Moreover, the interactions between HSP70 and HSP90, facilitated by co-chaperones like HOP and J-domain proteins, underscore the intricate network of molecular players involved in maintaining cellular proteostasis (38). The coordinated efforts of HSP70 and HSP90 and their associated co-chaperones ensure the proper folding, stabilization, and activation of diverse client proteins critical for cellular function (39). Understanding the distinct yet complementary roles of HSP70 and HSP90 sheds light on the sophisticated mechanisms governing PQC and cellular homeostasis in response to stress and environmental challenges (40).

### Co-chaperones

CHIP comprises three main functional domains: an N-terminal tetratricopeptide repeat (TPR) domain, a coiled-coil domain, and a C-terminal U-box domain (Fig. 2) (41). CHIP functions as an E3 ligase, which mediates the transfer of ubiquitin molecules from E2-conjugating enzymes to substrates through CHIP’s U-box domain. This ubiquitination event can lead to degradation, transportation, or sequestration of substrates (42, 43, 44). Ubiquitin-marked substrates can be recognized by the proteasome or lysosome and degraded. Thus, HSP70 interaction with CHIP’s TPR domain leads to decreased ATPase activity of HSP70 and the potential for ubiquitination and subsequent degradation of the chaperone-bound substrate (45). A similar mechanism occurs with HSP90 substrates (46).

HOP contains three tetratricopeptide repeat domain regions, each contributing to binding to either HSP70 or HSP90, two Asp-Pro (DP) rich domains, and nuclear localization sequences (Fig. 2) (47, 48). HOP aids in the folding of newly synthesized proteins and the refolding of damaged or misfolded proteins (49). The three TPR domains of HOP with differential binding affinity to HSP70 and HSP90 also allow HOP to function as a bridge for substrates to be handed off between the chaperones, placing HOP as an essential player in proteostasis (21, 50, 51).

Co-chaperones such as the CHIP and HOP bind to the c-terminal tail of HSP70 and HSP90 (52, 53). HSP70 and HSP90’s interaction with CHIP leads to increased ubiquitination and subsequent degradation of the substrates (53, 54); conversely, the binding of HOP to HSP70 and HSP90 complexes leads to substrate refolding (50, 54). HOP stimulates the ATPase activity of HSP70 and HSP90, providing more energy to fold substrates (50, 55). Conversely, CHIP inhibits the ATPase activity of HSP70 and minimally the C-terminus domain of HSP90, providing more opportunity to ubiquitinate the substrate (23, 45, 56, 57).

HSP40 proteins, called J-domain proteins, form a large family with over fifty members. These proteins feature highly conserved J domains that bind to HSP70, enhancing its ATPase activity as a co-chaperone (Fig. 2), stabilizing complexes between HSP70 and non-native proteins (58, 59, 60). This cooperative stimulation by HSP40 proteins is a general feature, as they act on different reaction steps to enhance the ATPase activity of HSP70 severalfold (60). HSP40 co-chaperones can modulate various functions of HSP70, such as determining client specificity, enhancing disaggregase activity, and affecting localization (61). HSP40 proteins play a crucial role in contributing to protein folding, stress responses, and maintaining cellular proteostasis.

### Consequences of a dysfunctional HSR

Dysregulation and accumulation of misfolded proteins in the cell are hallmarks of many human diseases, including cancer, Alzheimer’s, and cardiac disease. For example, hallmarks of Alzheimer’s disease include tau aggregates, a microtubule-associated protein that escapes degradation, and accumulation of p53 correlates with a poor prognosis in lung cancer (62, 63). Additionally, proteotoxic stress, the accumulation of proteins in the cell, is a stress hallmark of cancer (64). The PQC system, and the HSR in particular, plays an essential role in the initiation and progression of these diseases. In normal healthy cells, the HSR system quenches the protein levels of oncogenic proteins such as c-myc, a proto-oncogene encoding a transcription factor, mutant p53, a cell cycle control protein, vascular endothelial growth factor, epidermal growth factor receptor (EGFR), and human epidermal growth factor receptor 2 (65, 66, 67, 68, 69). Cancer cells can hijack this system by targeting tumor suppressor proteins for degradation or buffering the HSR system to cope with the proteomic stress induced by rapid cancer cell proliferation. Mutant p53, for example, can induce HSF1 transcriptional activity to promote the transcription of other oncogenic proteins such as Her2 and EGFR (70). In addition, multiple PQC system proteins are upregulated in cancer cells and mediate chemoresistance to chemotherapies (71). Conversely, increasing CHIP expression in renal cancer cells decreased cancer proliferation, highlighting an opposing role the HSR system can play in cancer proliferation (22). We recently reviewed the pro-*versus* anti-proliferative role of the HSR in cancer (72), and given the complexities of the involvement of the HSR in cancer, understanding key regulatory events may reveal the molecular underpinnings of certain cancers and identify new pharmacological targets.

Co-chaperone expression is associated with cancer progression, and, in some cases, causality has been established; however, context is essential. For example, higher CHIP expression is associated with a better cancer prognosis, as seen in the better tumor, node, metastasis stage, a staging system used by doctors to classify cancer progression and survival rates in some types of cancer, including renal and breast (42, 73). Alternatively, higher CHIP expression is also associated with a worse cancer prognosis, with higher tumor, node, metastasis stage and lower survival rates in glioma and gallbladder cancer (74, 75). Increased CHIP protein expression inhibited both renal cancer cell growth and breast cancer tumor growth in mouse studies, while CHIP knockdown increased renal cancer cell growth and breast cancer tumor growth (73, 76). Interestingly, HOP is upregulated in glioblastomas, pancreatic cancer, and breast cancer tumors (77, 78, 79, 80). Knockdown of HOP in glioma cells reduced proliferation rate and invasion through alteration to the protein kinase B or serine/threonine kinase 1 (AKT)/tumor necrosis factor receptor protein 1 (TRAP1) signaling pathway (77). HOP knockdown through RNAi in pancreatic cancer cells decreased the invasiveness of the cancer cells (78). Similar results were documented in other cancer cell line studies (81).

A pivotal element to protein triage in diseases lies in the balance between the pro-folding (HOP-associated) *versus* pro-degradation (CHIP-associated) environment. This switch between CHIP and HOP mediates the degradation of mutated, unwanted, or misfolded proteins like mutant p53 and, therefore, the outcome of diseases, which can ultimately provide new therapeutic targets. Specifically, PTMs on HSP70, HSP90, CHIP, and HOP offer a new point of regulation and insight into the mechanisms of cancer.

## Phosphorylation regulation of HSP70 and HSP90

### Protein phosphorylation

Protein phosphorylation adds a phosphoryl group (PO_3_) to specific side chains that alter the protein’s charge (54). This reversible modification typically occurs on serine, threonine, tyrosine residues, and histidine residues to a lesser extent (82). Kinase enzymes catalyze the transfer of the gamma phosphoryl group from ATP to the hydroxyl group of the side chain residue, a process reversed by phosphatase enzymes (82). The charge of the phosphoryl group affects allosteric binding between proteins. Phosphorylation is crucial in cell signaling propagation, enzyme regulation, and protein–protein interactions (Table 1). For example, phosphorylation affects HSP70 function, including co-chaperone interactions and the triage outcome of bound substrates (25).

**Table 1:** Key residues phosphorylated on HSP70 and HSP90 and the downstream effects of phosphorylation

Protein & phosphorylated residue	Modifier	Downstream impact	Preferred Co-Chaperone
HSP70			
Ser 385 (83)	ERK (83)	Chemo-resistance, Refolding	HOP
Ser400 (83)	ERK (83)	Chemo-resistance, Refolding	HOP
Thr636 (22)	AKT (88, 96)	Chemo-resistance, Refolding, and Increased Proliferation	HOP
Ser634 (22)		Refolding	HOP
Ser631 (88, 108)	AKT (88)	Decreased mitosis	HOP
Ser633 (108)	AKT (88)	Decreased mitosis	HOP
HSP90			
Thr725 (22)		Decreased proliferation	HOP
Ser726 (22)		Decreased proliferation	HOP
Thr90 (93)	PKA (93)	Increased degradation	CHIP
Thr5/Thr7 (111)		Increased chemo-response	CHIP
Thr115/T425/Thr603 (94)		Increased cell migration	HOP

Numbers in parentheses refer to references for papers that describe the modifications. All modifiers have been referenced accordingly.

### Phosphorylation of HSP70 in the NBD and linker domain

Phosphorylation of HSP70 at Ser385 and Ser400 by extracellular signal-related kinase (ERK) increased the refolding activity of HSP70 about 1.5-fold (83). Additionally, a phospho-null mutant of HSP70 at Ser385 and Ser400 sites showed increased ubiquitination of client proteins AKT and cyclin-dependent kinase 4. In contrast, a phospho-mimetic of those same sites had a decrease in the ubiquitination of client proteins (22, 83, 84). hYVH1 is a phosphatase that interacts with HSP70 and enhances cell survival after heat shock (85). Other kinases involved in HSP70 phosphorylation include polo-like kinase 1, PKA, and AKT (86, 87, 88).

### Phosphorylation of HSP90 outside the CTD

Many studies have reported on HSP90 phosphorylation sites (89, 90, 91). PKA can phosphorylate HSP90 and allows for interaction with Cdc37, a cell cycle protein (92). However, phosphorylation *via* PKA at Thr90 increases the association with CHIP while decreasing the association with HOP (93). The Thr90 phosphorylation site also reduces the affinity for ATP to bind to HSP90⍺, decreasing the chaperone refolding cycling (93). Protein kinase Cγ (PKCγ) phosphorylates HSP90⍺ at multiple threonine residues Thr115/Thr425/Thr603, decreasing chaperone activity (94).

### Phosphorylation of HSP70/90 tail influencing CHIP and HOP binding

Many studies have highlighted the C-terminus EEVD motif of HSP70, specifically PTMs on this motif, is vital in substrate fate outcome. The EEVD motif is where many co-chaperones interact with HSP70, making it a critical regulation point to determine co-chaperones associated with HSP70 (95). When unphosphorylated on the c-terminus domain at Thr636, HSP70 preferentially binds to CHIP, promoting the degradation of chaperone substrates, as seen in renal cancer (Fig. 2) (22). However, when phosphorylated at Thr636 and Ser634, HSP70 preferentially binds to HOP, decreasing the degradation of those downstream proteins in renal cancer (22, 96). Assimon *et al.* and Zemanovic *et al.* also highlighted the impact of phosphorylation on chaperone binding, specifically those that bind using a TPR domain, such as CHIP and HOP (88, 97). A pseudo-phosphorylated EEVD motif in the c-terminus domain of HSP70 altered the polarization binding between CHIP and the motif. It decreased the binding affinity constant, indicating a change in affinity for the pseudo-phosphorylated tail (97). Phosphorylation of Ser631, still on the tail of HSP70, impacts the association of CHIP, as seen in a decreased pull-down of CHIP in co-immunoprecipitation immunoblotting (88). Phosphorylation of HSP90 that occurs predominantly on its c-terminal tail affects its binding and interaction with co-chaperones (Fig. 2). Phosphorylation at Thr725 or Ser726 increases the affinity between HSP90⍺, one of four isoforms of HSP90, and HOP (22). In contrast, HSP90⍺ has a higher affinity for CHIP in its unphosphorylated form (22).

## Phosphorylation regulation of CHIP and HOP

### CHIP phosphorylation

Protein kinase G (PKG) phosphorylates CHIP at Ser19 and enhances its interaction with HSP70 *via* stabilizing CHIP’s half-life, improving its resistance to degradation (98). Ser19 phosphorylation also did not affect the ATPase activity of HSP70 (98), suggesting that Ser19 phosphorylation mainly functions through enhancing CHIP stability, supported by the finding that the phospho-null mutation at Ser19 decreased CHIP’s interaction with HSP70. Phosphorylated CHIP reduced proteotoxicity and more effectively cleared ubiquitinated proteins (98). CHIP can also be phosphorylated by cyclin-dependent kinase 5 at Ser19 (Ser20 in mice), causing a decrease in interaction between CHIP and truncated apoptosis-inducing factor in neuronal cells (99). This decreased interaction induces neuronal death, highlighting the essential role of CHIP in cell death regulation (99). Aurora kinase A phosphorylates CHIP at Ser273, enhancing ubiquitination by CHIP of androgen receptors (ARs), which are essential regulators involved in prostate cancer (100). A phospho-null CHIP Ser273 mutant attenuated AR degradation compared to WT CHIP, indicating that CHIP phosphorylation activates ubiquitin towards ARs (100). Since ARs are targets for prostate cancer treatment, increasing CHIP phosphorylation and, therefore, the HSP70-CHIP association could be a target for prostate cancer treatment. ***Current published data on CHIP phosphorylation*** (Table 2) ***focus on the interaction between HSP70 and CHIP, but gaps in knowledge remain regarding the regulatory impact on HSP90***. However, based on the similarities in the interaction between HSP90 and HSP70 with CHIP, we predict that the phosphorylation of CHIP would have a similar effect on its association with HSP90.

**Table 2:** Key residues phosphorylated on CHIP and HOP and the downstream effects of phosphorylation

Phosphorylated residue	Modifier	Downstream impact
CHIP		
Ser19 (98)	PKG (98)	Increased chaperone interaction
Ser19 (99)	CDK5 (99)	Increased neuronal death
Ser273 (100)	Aurora kinase A (100)	Increased ubiquitination activity
HOP		
Ser189 (102)	Casein kinase II (101)	Nuclear translocation
Thr198 (102)	Cdk1 (101)	Nuclear exclusion
Thr37 (103)		Decreased activation activity
Ser95 (103)		Decreased activation activity
Tyr354 (104)		Decreased co-chaperone activity

Numbers in parentheses refer to references for papers that describe the modifications. All modifiers have been referenced accordingly.

### HOP phosphorylation

On the competing side of the HSP70/90 co-chaperone switch, HOP can be phosphorylated by cyclin-dependent protein kinase 1 (Cdk1) and the protein kinase casein kinase II (CK2) at multiple sites that impact function (Table 2) (101). Ser189 phosphorylation causes a nuclear translocation of HOP and mediates the assembly of the HSP70 chaperone complex (102). However, phosphorylated HOP-Thr198, or a phosphomimetic HOP at Thr198, is restricted from the nucleus, compared to the phospho-null at Thr198, further demonstrating that PTM location dictates protein localization (102). Phosphorylation of the TPR1 domain of HOP on Thr37 and Ser95 decreased glucocorticoid receptor activation activity and reduced affinity for HSP70 (103), and the unphosphorylated Tyr354 of HOP is required for optimal HSP70 substrate accumulation and activity (104). Because it promotes protein folding and cell cycle progression, decreasing HOP expression is a target for cancer therapies. As mentioned above, HOP is highly expressed in tumors, including glioblastomas, pancreatic cancer, and breast cancer (77, 78, 79, 80). Pharmacologically altering the activity state of HOP through phosphorylation could decrease cancer proliferation and improve prognosis. Shifting the preference away from HOP toward CHIP–HSP70 and CHIP–HSP90 complexes may benefit when HOP is aberrantly expressed at high levels.

## Impact of phosphorylation on cancer cell proliferation

### Shifting co-chaperone binding

Depending on the co-chaperone complex, HSP70 and HSP90 can promote or prevent cancer cell proliferation. Promoting HSP70–CHIP interactions inhibited tumor necrosis factor-⍺–induced apoptosis by degrading apoptosis signal-regulating kinase 1 (105). Additional downstream substrate degradation mediated by CHIP–HSP70 interactions influences cancer progression. For example, c-myc, a common oncogenic protein mentioned above, is regulated by heat shock in cancer cells (65). Blocking CHIP ubiquitination of HSP70 through blocking the BCL2-associated athanogene 2–CHIP interaction induces apoptosis in gastric cancer, decreasing the CHIP–HSP70 interaction and allowing for a better prognosis in this cancer (106). A phospho-mimetic of HSP70 at Thr636 mimicking natural phosphorylation, reduced binding with CHIP, and increased cellular proliferation in HEK293 cell lines.

In contrast, a phospho-null mimetic has the opposite effect, increasing CHIP binding and decreasing cellular proliferation (22). Muller *et al.* found higher amounts of C-terminal tail phosphorylation on HSP70 (Thr636) in breast tumor samples compared to normal tissue that coincided with increased expression levels of HOP (22). Phosphorylation of HSP70 at Ser385 and Ser400 also increased the cell viability and resistance to HSP70 inhibitor–mediated cell death in HEK293T cells, another cancerous cell line (83). Phosphorylation of the Tyr288 site on HSP70 regulates the transportation of methotrexate, a chemotherapeutic agent, into leukemia cells, affecting these cancer cells’ resistance to methotrexate (107).

### HSP70 function and activity

HSP70 and its PTMs play crucial roles in maintaining proteostasis by regulating various cellular functions. However, these mechanisms can become dysregulated during tumor progression. Phosphorylated HSP70 at Ser631 and Ser633 localizes to the centrosome during mitosis and induces mitotic spindle elongation and mitotic arrest (108). This phosphorylation prevents cells arrested in mitosis by arsenic trioxide from apoptosis (108). However, O’Regan *et al.* indicated that HSP72, a cytoplasmic isoform of HSP70, is phosphorylated by Nek6 at Thr66, and phosphorylated HSP72 promotes cell cycle progression, specifically highlighting the importance of HSP72 in regulating cancer cell division (109). Disruption of the cell cycle often leads to uncontrolled cell growth, a hallmark of cancer, highlighting PTMs of HSR as a critical point of regulation and control. Without HSP72, HeLa cancer cells could not align chromosomes and, therefore, properly divide, causing an increase in multinucleated cells and chromatin bridges, indicating mitotic progression breakdown (109). This data highlights a potential target to inhibit cancer cell cycle progression.

### HSP90 function and activity

PTMs of HSP90 control its function in healthy cells, but these modifications also influence cancer cell proliferation and tumor progression. Phosphorylation of HSP90 is linked to enhanced chaperone function and many cancer-driving processes (24, 91). Phosphorylated HSP90⍺ at Thr725 and Ser726 decreased doubling time in HEK293 cells, while the phospho-null form of HSP90⍺ at those same sites increased the doubling time of the cells, indicating a decrease in proliferation (22). Additional immuno-blotting comparing breast cancer tissue samples with normal tissue samples showed an increase in phosphorylated HSP90*α* in the breast tissue (22). Regulation of these phosphorylation events impacts HSP90*α*′s role in cancer proliferation. In a reciprocal regulation loop, HSP90⍺ prevents PKCγ degradation and facilitates PKCγ translocation to the membrane, activating its kinase activity and promoting cell migration and proliferation (94). Inhibition of both phosphorylation of HSP90⍺ at Thr115/Thr425/Thr603 by PKCγ and PKCγ itself prevented cell migration and induced apoptosis in colon carcinoma cells (94). An upregulation of phosphorylated HSP90β on Ser254 was seen post-anticancer treatment with 5-fluorocytosine, a prodrug used in tumor gene therapy treatment, highlighting that HSP90β might contribute to cancer regression (110). Further studies with HSP90*α* showed the phosphorylated HSP90⍺ at Thr5/Thr7 correlated with a good response for patients to platinum-based chemotherapy. They highlighted a potential use of HSP90⍺ as a prognostic marker for this type of chemotherapy (111).

Altering the phosphorylation of HSP70 and HSP90 impacts many downstream processes (Table 1), including interactions with CHIP and HOP, ultimately influencing cancer prognosis. The data showcased in most cancer systems suggest the increased association of HSP70 and HSP90 with HOP is pro-proliferation; therefore, targeting a switch in preference to CHIP-containing chaperone complexes may have anticancer benefits, highlighting the role for balance between CHIP- *versus*HOP-containing chaperone complexes. Other research has focused on co-chaperones as a point of control and revealed how CHIP and HOP can also be phosphorylated, ultimately affecting their association with HSP70/90, a topic we explore below.

## Impact of other PTMs and components

Additional PTMs of the HSP70/co-chaperone and HSP90/co-chaperone complex impacting protein–protein interactions and function include acetylation, methylation, ubiquitination, glycosylation, and nitration.

### Acetylation

Protein acetylation is the addition of an acetyl group onto side chains, catalyzed by acetyltransferases (112). This modification can be reversible or irreversible, thus impacting the long-term and short-term functional consequences. Acetylation occurs predominantly on lysine residues and, to a lesser extent, on alanine, arginine, serine, and threonine residues (113).

Acetylation of HSP70 influences the binding preference of co-chaperones to HSP70 and can, therefore, dictate the result of protein triage. After the onset of stress, arrest-defective one protein (ARD1) acetylates HSP70 at Lys77, which pushes the preference of binding towards HOP, leading to the refolding of substrates (114). HSP70 is deacetylated in the later stages of the stress response, shifting the binding preference to CHIP (114). Without this acetylation, the cellular stress response is limited, and damaged cells do not undergo apoptosis, perhaps promoting cancerous cell formation. Acetylation at Lys126 also attenuates the chaperone activity of HSP70 by changing the physiological function of HSP70 and altering co-chaperone binding. This site increases HSP70’s association with CHIP over HOP, thus creating a pro-folding, pro-tumor environment (115). Acetylation of HSP70 at Lys126 inhibits MDA-MB-231 cancer cell proliferation, tumor cell invasion, and migration in breast cancer cells (115).

In addition to the HSR mechanism, HSP70 ensures cellular health by regulating chaperone-mediated autophagy and apoptosis. Yang *et al.* found that acetylated HSP70 is required to adequately form the autophagosomes that break down the cellular components for recycling (116). Specifically, acetylated HSP70 regulates Vps34, also known as the kinase PIK3C3, and its association with Beclin 1, a regulator of the autophagy pathway, marking a pivotal step in autophagosome formation (116). Acetylation of HSP70 at Lys126 increases the sequestering of Bcl2, a mitochondrial membrane protein that blocks cell death, for autophagosome formation (115). Additionally, this acetylation site prevents HSP70 from binding with apoptotic peptidase activating factor 1 and allograft inflammatory factor 1 and, therefore, mitigates the promotion of apoptosis (115). Without the ability to induce cell death, accumulating unhealthy cells could lead to cell transformation.

Acetylation of HSP90 also affects ATP binding, client binding, and co-chaperone activity (24). HSP90 is acetylated at multiple sites in the middle and CTDs, but the most influential factor affecting the co-chaperone role is at Lys294, between the charged linker region and the middle domain (Fig. 2) (117). When acetylated, HSP90 binds less CHIP, confirming that acetylation influences HSP90’s change in activity and interaction with co-chaperones (117). HSP90 is deacetylated by histone deacetylase 6 (HDAC6) (117, 118), and the inactivation of HDAC6 results in the hyperacetylation of HSP90, a loss of chaperone function, and dissociation with p23, a protein essential to glucocorticoid receptor maturation (118). ***Investigating this acetylation event in a cancer context could provide new insight and opportunities for treatment.***

### Methylation

Protein methylation by methyltransferases is a reversible PTM that occurs mainly in the nucleus, with histones being one of the most studied substrates for methylation and critical for transcriptional regulation of the genome. Methylation often occurs on lysine and arginine residues, significantly altering the target protein's structure and affecting its function (113). Methylation of HSP70 at Lys561 by HSPA- KMT, a lysine methyltransferase that methylates the specific residues in multiple HSP70 (HSPA) proteins, has a growth-promoting effect in HeLa cancer cells by enhancing Aurora kinase B activity (119, 120). Clinical tissue samples from bladder and liver tumors mirror this cancer influence where the dimethylation of Lys561 of HSP70 is significantly elevated in these cancer tissues (120). The methylation of HSP90 at Lys607 affects the closed confirmation of HSP90, changes ATP binding activity, and decreases Aha1 binding, a co-chaperone activator of HSP90 ATPase, ultimately altering client activation (121).

### Ubiquitination, glycosylation, and nitration

There are limited reports on ubiquitination, glycosylation, and nitration, influencing co-chaperone association and subsequent cancer progression. Ubiquitination is the addition of ubiquitin, a small 8.5 kDa protein, onto a side chain of a protein, predominantly lysine residues, catalyzed by a cascade of three enzymes, E1, E2, and E3 (113). Events include single ubiquitination reactions (mono-ubiquitination) or polyubiquitination of any one of the seven lysine residues on ubiquitin, resulting in polyubiquitin chains. The result of ubiquitination alters the structure and interactions of the substrate protein. Ubiquitination is a critical signaling event in PQC. The 26S proteasome recognizes specific polyubiquitin chain topologies, and this interaction leads to the degradation of the ubiquitinated protein and recycling of the protein building blocks (122). CHIP can ubiquitinate HSP70 itself or substrates interacting with HSP70, leading to its degradation, but requires the HSP70-lid domain, positioned right before the EEVD motif in the CTD (Fig. 2), for proper ubiquitination of either (84, 123). ***Investigation of the functional effect of ubiquitinated HSP70 beyond degradation has not been reported*** (84, 124). Poly-ubiquitination of HSP90 at multiple sites (Lys6, Lys11, Lys48, and Lys63) leads to its degradation (125).

Although less studied, protein glycosylation and nitration can impact the function of proteins. In glycosylation, oligosaccharide chains are linked to residues through covalent bonds, affecting interactions structurally (113). The subsequent effect on protein–protein interaction depends on the specific type of glycosylation: O-linked, N-linked, or C-linked. ***However, it is known that HSP90 is glycosylated on the four ⍺-subunits and S434 and S461 of HSP70β, but few studies have explored the effect of glycosylation on the function of HSP90*** (126). Nitration adds a nitro group to tyrosine residues of proteins and changes the proteins’ charge (127). The nitration of HSP90 at Tyr33 and Tyr56 induces cell death by activating the Fas pathway, a type of programmed cell death that activates apoptosis and is a toxic gain of function that turns HSP90 into a toxic protein (128). Triggering cell death *via* the Fas pathway by the nitration of HSP90 may lead to new cancer therapeutic approaches (128).

***Despite many studies investigating the points of other PTMS, there is a lack of published studies on the effect of these PTMs on co-chaperone binding, highlighting a critical gap in knowledge***. Based on the growth-promoting effects of these PTMs, it is predicted that the preference is for HOP in the cancer environment, highlighting a potential point of treatment.

### Heat shock factor 1

HSFs include nucleotide-binding, regulatory, and CTDs (Fig. 2) (129). They regulate the transcriptional response to heat or stress (8). When faced with a stressor, HSFs dissociate from their inhibitory complex with HSP90 and bind to DNA to induce the transcription of numerous genes, including those that encode HSP70 and HSP90 (8). Early research on HSF1 implicated that CHIP is required for HSF1 dimerization and entry into the nucleus, promoting a feed-forward regulator mechanism to ramp up the HSR to alleviate proteostatic stress (130). PTMs of HSF1 alter its ability to bind and associate with DNA and other HSPs, modulating the cellular response (Table 3).

**Table 3:** Key residues phosphorylated on HSF1 and the downstream effects of phosphorylation

Phosphorylated residue	Modifier	Downstream impact
Ser326 (135)	AKT1 (135)	Increased transcriptional activity
Thr142 (135)	AKT1 (135)	Increased trimerization
Ser230 (135)		HSR activation
Ser326 (135)		HSR activation
Thr527 (135)		HSR activation
Ser303/Ser307 (136)		Decreased transcriptional activity
Ser216 (139)	PLK1 (139)	Cell cycle progression
Ser419 (138)	PLK1 (140)	Increases transcription
Thr120 (141)		Increases proteostasis

Numbers in parentheses refer to references for papers that describe the modifications. All modifiers have been referenced accordingly.

Manipulating PTMs of HSF1 can inhibit cancer cell proliferation and susceptibility to chemotherapeutics. Multiple signaling pathways in cancer cells activate HSF1 to accommodate the rapidly changing tumor environment and stress of rapid cell proliferation (131). HSF1 is frequently overexpressed in cancer cells (132, 133). As mentioned above, HSF1 overexpression inhibited mitotic exit, causing an increase in aneuploidy and multinucleated cells, a hallmark of cancer (132). Additionally, HSF1-mediated aneuploidy was accelerated in p53-defective cells, a defect frequently seen in cancer cells (134). Therefore, decreasing HSF1 expression to lower HSP70 and its interaction with HOP would decrease cancer progression and thus make HSF1 a promising target for chemotherapy.

#### Phosphorylation

AKT phosphorylates and activates HSF1 (135). Specifically, AKT phosphorylation of Ser326 increases HSF1’s transcriptional activity, while phosphorylation at Thr142 is necessary for HSF1 trimerization (135). Other sites, such as Ser230, Ser326, and Thr527, are required for maximum activation of HSF1 during heat stress (135). Constitutive phosphorylation at Ser303 and Ser307 negatively regulates HSF1 transcription activity at physiological temperature, highlighting an essential step in HSF1 regulation (136). Cyclosporin A (CsA), an immune suppressant, enhances the phosphorylation of these sites (Ser303 and Ser307) and inhibits HSF1’s transcriptional activity and, thus, the ability to respond to heat shock (137). CsA also interferes with HSF1 complex formation, nuclear translocation, and binding to the HSP70 promoter (137). PLK1 also phosphorylates HSF1 at multiple sites, including Ser216 and Ser419 (138, 139, 140). Phosphorylation of HSF1 at Ser216 occurs during the early stages of mitosis and propels mitosis forward through interaction with Cdc20, a cell cycle regulatory protein (132, 139). Without the phosphorylation and subsequent binding to Cdc20, mitosis does not continue (132). However, too much HSF1 inhibits mitotic exit, leading to aneuploidy and multinucleated cells (132). The Ser419 phosphorylation site of HSF1 helps recruit the TRRAP–TIP60 acetyltransferase complex to promoters (138). This recruitment changes histone modifications, especially on the HSP72 promoter, and opens chromatin for transcription (138). Phosphorylation of HSF1 at Thr120 protects HSF1 from being ubiquitinated by F-box and WD repeat domain containing 7 (FBXW7), a tumor suppressor protein of the F-box protein family, and subsequently degraded (141, 142). This HSF1 Thr120 phosphorylation promotes proteostasis and binding to the programmed death ligand 1 (PD-L1) promoter (141).

#### Acetylation and ubiquitination

Although less reported than phosphorylation, acetylation and ubiquitination contribute to the post-translational landscape of HSF1 regulation. SIRT1, a well-characterized sirtuin family member, deacetylates HSF1 at Lys80 to induce the expression of other HSR proteins, such as HSP70 (143, 144). Acetylation negatively affects the DNA-binding ability of HSF1 (144). Acetylation by EP300, a transcriptional co-activator protein, at other sites, such as Lys118, Lys208, and Lys298, stabilizes the expression of HSF1 as it prevents ubiquitination and subsequent degradation (13, 145). FBXW7, a critical tumor suppressor, ubiquitinates HSF1 and leads to HSF1 degradation (142, 146). Without FBXW7, HSF1 accumulates and activates the metastatic pathway of melanoma, driving cancer survival and metastasis (142). Therapies could target these PTMs involved in HSF1 stability or activity.

Most work on HSF1's PTMs has focused on its nuclear activity as a transcription factor. However, HSF1 does have some role in the cytosol, where it interacts with CHIP. ***Investigating the roles of PTMs on HSF1 in the cytosol would provide a better understanding of HSF1’s role in HSR and cancer progression and reveal if there are cytosolic ramifications of HSF1 PTMs.***

### Heat shock protein 40

Another key player in the HSP70/co-chaperone complex is the HSP40 family of co-factor proteins, also known as J-domain proteins, that shares a conserved J domain on the N-terminus of the protein and includes cysteine string proteins and DNAJBs (Fig. 2) (147). Other domains in this protein include Zinc finger motifs and a CTD (Fig. 2) (148). HSP40s bind proteins and target them to HSP70 for folding and enhance the ATPase activity of HSP70s (147, 148). The primary functions of HSP40 are to increase the ATP hydrolysis rate of HSP70s, engage polypeptide substrates to HSP70s, and promote a profolding environment (149). CHIP can inhibit HSP40 by blocking the binding of HSP40 to HSP70 and, subsequently, the HSP40-stimulated activity of HSP70 (45). CHIP acts as a modulator of HSP40 to pause the ATPase activity of HSP70, promote substrate ubiquitination, and cause a shift to pro-degradation. HSP40 proteins, like the rest of the HSPs, undergo PTMs that contribute to the acute regulation of the heat shock mechanism and stress response. Manipulating HSP40s may impact the profolding *versus*pro-degradation associations of HSP70 and HSP90 with HOP *versus*CHIP, respectively.

HSP40s can be phosphorylated by multiple kinases, including mitogen-activated protein kinase–activated protein kinase 5 (MK5) and CK2, or can even exist as phosphoproteins in the cell (147, 150, 151). When phosphorylated by MK5 at Ser149, Ser151, and Ser171, HSP40-mediated regulation of HSF1 activity increases, allowing for a more robust stress response (150). Phosphorylation on Ser10 of the cysteine string protein triggers a conformational switch in the protein’s orientation and activity and plays a role in presynaptic maintenance by preventing neurodegeneration, highlighting the crucial role of HSP40s in cellular viability (152, 153, 154).

The DNAJB-PRKACA gene fusion, created by the fusion of a subunit of the HSP40 complex (DNAJB) and the catalytic subunit of protein kinase A (PRKACA), creates a fusion kinase and drives fibrolamellar hepatocellular carcinoma, and PTMs of this kinase could impact cancer progression (155). DNAJB1 has four phosphorylation sites in its cytosolic domain that alter its nuclear transport (151) and directly affect DNAJB1’s role in cancer progression (156). DNAJB enhances the ubiquitination of MIG6, a tumor suppressor, and promotes EGFR signaling (157). When DNAJB was knocked down, lung cancer cells became sensitized to treatment with gefitinib, a common chemotherapeutic targeting tyrosine kinases (157). HEDJ, an HSP40 co-chaperone, is localized to the endoplasmic reticulum and can be glycosylated (158), ***although the potential implications of this glycosylation on cancer progression have not been reported.***

### Small HSPs

Small heat shock proteins (sHSPs) are molecular chaperones between 15 to 30 kDa and have a conserved sequence of 80 to 100 amino acids, essential for the function of sHSPs (Fig. 2) (159). sHSPs also have a conserved N-terminal region and a short sequence at the c-terminus (160). sHSPs include HSP beta-2, HSP17, HSP20, clusterin, and HSP17, all of which aid in the formation and folding of proteins by assisting chaperones (160, 161). However, only HSP27 is post-translationally modified, so we focus mainly on HSP27 (159). HSP27 can be phosphorylated on different regions by multiple kinases, including p38 mitogen-activated protein kinase (MAPK), MAPKAPK-2, another protein kinase in the MAPK family, AKT, and PKG (162). HSP27 is upregulated in many cancers, such as prostate, lung, and pancreatic (163, 164). Therefore, targeting HSP27, specifically phosphorylation of HSP27, has been promising for cancer therapies.

HSP27 phosphorylation is associated with cancer progression (165). Phosphorylation of HSP27 at Ser15, Ser78, or Ser82 initiates chemoresistance in prostate cancer cells (166). Overexpression of HSP27 led to increased survival rates of prostate cancer cells after docetaxel, a microtubule-targeted chemotherapy treatment, while the overexpression of the nonphosphorylatable HSP27 mutant decreased resistance to treatment (166). Similarly, overexpression of the same nonphosphorylatable HSP27 mutant with gemcitabine, a DNA replication–targeted chemotherapy treatment in pancreatic cancer cells, decreased cell proliferation (164, 167). Preventing phosphorylation of HSP27 by p38 MAPK and MAPKAPK-2 inhibition increased cell death of pancreatic cancer cells after treatment with gemcitabine (164, 167). Phosphorylated HSP27 at Ser15, Ser78, or Ser82 also contributes to the chemoresistance of adenocarcinoma cells (168). Overexpression of HSP27 phospho-mimetic mutants activated p53 and decreased cell death (168). Inhibition of this same phosphorylation in combination with doxorubicin decreased p53 induction and increased cell apoptosis (168). Because HSP27 promotes the folding and refolding of substrates, one would predict it preferentially associates with HOP. ***However, further studies would be needed to confirm that.***

## Advances in drug therapies

Our literature review suggests that the balance between a pro-degradation environment and a profolding environment can influence cancer cell proliferation, transformation, and resistance to chemotherapy. Consequently, developing drugs that can modulate the HSR and the balance between protein degradation and folding could be a promising approach for cancer therapy. This modulation could be achieved by directly targeting the proteins involved in the HSR or the upstream enzymes, such as kinases and acetyltransferases.

### HSP70 and HSP90

#### Direct-acting drugs

HSP70 has already been shown to be a promising pharmacological target (169). Many studies investigated small molecules that target HSP70 function, summarized in a review by Nitzsche *et al.* (170). For example, the novel inhibitor for HSP70 named S1g-6 from Wang *et al.* showed selective cell death in cancer cells over normal cells (171). However, as mentioned above, PTM specificity determines the influence on cancer progression. For example, methylation of HSP70 has a growth-promoting effect, while acetylation inhibits cancer proliferation (115, 119). HSP70 plays a role in regulating apoptotic pathways promoting cancer progression, but it has also been shown to induce an immune response to target cancer cells, indicating a potential dual role in cancer progression (172). ***The duality of HSP70 is a crucial consideration for cancer therapies, and characterizing and specifying HSP70 substrates as pro-versus anti-cancer HSP70 is essential for developing cancer treatments.***

Because of its prominent role in the HSR, HSP90 is another promising drug target for cancer treatment, with multiple drugs and compounds shown to decrease cancer cell proliferation that moved into clinical trials (34, 92, 173). One example is 17-AAG and its derivates, which showed anticancer activity in small-cell lung cancer, but the trials were stopped before reaching phase III due to safety concerned and limited efficacy (92). Another HSP90 inhibitor currently in clinical trials is ganetespib, a small-molecule inhibitor targeted at the ATP-binding site of HSP90 (174). In phase II trials, non-small cell lung cancer showed a response to ganetespib. However, no response was seen in refractory metastatic colorectal cancer, again highlighting a critical consideration when targeting the HSR system, which depends on the specific role and PTMs of the component that will determine the effectiveness of inhibitors (29). ***Further exploration of specific inhibitors for HSP90 is needed to ensure anti-cancer treatment rather than pro-cancer results.***

Drugs targeted to the tail of HSP70/90 could significantly impact the pro-degradation and pro-folding environment mediated by CHIP and HOP occupancy. However, limited trials on compounds target the PTMs in the tail region of HSP70/90. Nadler *et al.* isolated a compound named DSG that targeted the EEVD motif of HPS70 and enhanced its steady-state ATPase activity (175). DGS showed antitumor properties but did not pass phase II trials (176, 177). HVH-2930 targets the CTD of HSP90, affecting the ATP-binding pocket of the HSP90 dimer and inhibiting its activity (178). Bhatia *et al.* also created a small-molecule inhibitor, 5b, that targeted the CTD of HSP90 and showed anti-leukemia properties (179). ***The impact of HVH-2930 and 5b on HSP90 PTMs and CHIP/HOP occupancy in these two molecules was not reported.*** However, targeting the CTD of HSP90 could shift CHIP- and HOP-binding dynamics and cancer progression.

#### Kinases and acetyltransferases

One approach to targeting PTMs is to drug the upstream enzymes, such as kinases and acetyltransferases. As mentioned above, ERK phosphorylates HSP70. Current progress on ERK inhibitors is limited, but a few are in clinical trials (180). Ulixertinib has shown promising anticancer effects in pancreatic cancer models (181). However, inhibiting these enzymes could be nonspecific to the HSR, as these enzymes target multiple other proteins and could have many adverse or opposite effects on cancer progression (182).

PKC*γ* is a kinase that targets HSP90 along with many other proteins. Although there are multiple places of inhibition on the PKC protein, most are targeted to the C3 domain, but some bind to the C1 domain. The compound UCN-01 showed promising results in multiple phase I drug combination therapies for cancer treatment, but few combinations made it through phase II (183). The upstream ORF peptide upstream of PKC inhibits the catalytic activity of PKCs and inhibits cancer cell survival, progression, and metastasis (184). Specifically, inhibiting PKC in non-small cell lung cancer has been a promising therapeutic focus to prevent resistance; however, many trials of PKC inhibitors have shown little clinical benefit (183, 185).

PKA is one of the kinases that phosphorylate HSP90. Endogenous PKA inhibitor (PKI) proteins such as PKIα, PKIβ, and PKIγ bind with PKA and inhibit its kinase activity. Prostate cancer cells show less migration and tumor growth when these inhibitor proteins are knocked down (186). However, like the rest of the HSR system, PKA signaling can be pro- and anti-tumor growth (187).

ARD1 and HDAC6 are the acetyltransferases responsible for acetylating HSP70. ARD1 genetic inhibition reduced the levels of NRF2, a critical oncogenic transcription factor protein in colon cancer cell lines, highlighting a protein role in cancer therapy (188). shRNA knockdown of ARD1 also inhibited prostate tumorigenesis (189); however, specific ARD1 inhibitors have yet to be available for clinical trials. HDAC6, on the other hand, has many promising inhibitors that enhance the effectiveness of different chemical therapies (190). The small molecule inhibitor ACY1215 targeted to HDAC6 reduced tumor growth rate in non-small lung and gallbladder cancer cells (191, 192). Therefore, HDAC6 could be therapeutically beneficial for cancer treatment.

### CHIP and HOP

#### Direct-acting drugs

Although there are no current drug therapies in clinical trials that target CHIP currently, many studies have alluded to inhibiting CHIP as a mechanism of action to prevent cancer cell proliferation. Inhibiting CHIP in both colorectal and prostate cancer, where CHIP acts as a tumor promoter, decreases cell growth, migration, and invasion (193, 194). However, as highlighted in previous reviews, CHIP plays a dual role as a tumor suppressor and oncogenic protein (42, 72, 195, 196). Overexpression of CHIP decreased renal cancer cell growth, and the suppression of CHIP promoted ovarian cancer metastasis, both instances where CHIP acts as a tumor suppressor (73, 197). Therefore, a better understanding of its role in different cancer types is needed before any drugs are targeted to CHIP.

The HSP70 inhibitor VER155008 suppresses the expression of HOP, which decreases prostate cancer growth (198). HOP was implicated as a prognostic biomarker in ovarian cancer and breast cancer, and when knocked down, pancreatic, colorectal, and breast cancer cell progression decreased, highlighting how targeting HOP and likely shifting the binding occupancy of HSP70/90 to HOP and CHIP could be beneficial for cancer treatment (78, 80, 199, 200). A compound generated by Pimienta *et al.* inhibited the HOP–HSP90 complex and demonstrated anticancer effects in breast cancer cell lines (201). However, further studies on this compound or others targeted towards HOP have yet to be published.

#### Kinases and acetyltransferases

Activation of PKG, the kinase that phosphorylates CHIP, is implicated in multiple cancer studies as a target to decrease cancer cell progression (202, 203). However, other studies have indicated that the nitric oxide pathway, containing PKG, protects against apoptosis in ovarian cancer cells and increases the stemness and metastasis in lung and breast cancer cells (204, 205). Compound KT5823 inhibits protein kinase activity *in vitro* but did not show any effects in human platelets or rat mesangial cells (206, 207). As indicated above, CHIP also has a complicated, dual role in cancer progression; therefore, the dual role of PKG does correlate with its influence on CHIP.

Aurora kinase A (AURKA) is another kinase that phosphorylates CHIP. It has been implicated as a synthetic lethal target for many other tumor suppressors. Targeting AURKA and other tumor suppressors results in cancer cell death, highlighting a promising target for cancer treatment (208). Clinical trials of AURKA molecular inhibitors are currently underway (209).

Targeting the kinases and acetyltransferases that modify HOP to decrease its association with HSP70/90 is another promising strategy to reduce cancer progression. CDK1 is the kinase that phosphorylates HOP. Many inhibitors targeted to CDK1 have shown promising results in decreasing cancer cell progression and have entered clinical trials (210). Specifically, a few novel CDK1 inhibitors from Akl *et al.* had antiproliferative activity in a panel of pancreatic cancer cell lines (211). Other reviews have highlighted the impact of CDK1 inhibitors in breast cancer, emphasizing that inhibiting HOP phosphorylation would be promising to decrease cancer cell progression (210, 212). CK2 is another kinase that can target HOP for phosphorylation. Inhibitors targeted towards this kinase have been implicated in treating myeloid leukemia and triple-negative breast cancer, although there have been limited clinical effects in leukemia treatment (213, 214, 215).

### Heat shock factor 1

Recent studies detail HSF1 pharmacology, especially regarding its phosphorylation status. AKT phosphorylates HSF1 independent of heat stress in breast cancer cells (216). Inhibition of AKT and, therefore, inhibition of HSF1 phosphorylation reduced the proliferation of PIK3CA mutated cells, a common mutation in the gene that makes the PI3K enzyme (216). Biochemical inhibition or siRNA silencing of HSF1 increased benzimidazole carbamate drug potency in colorectal cancer cells (217). Further experiments found that ERK-1/2 activates HSF1 through phosphorylation of S326 and promotes chemotherapeutic resistance in colorectal cancer cells (217). Bortezomib, a proteasome inhibitor for treating multiple myeloma and mantle cell lymphoma, induces cancer cell apoptosis and cell cycle arrest by blocking the 26S proteasome, which also disrupts DNA replication and repair (218). Bortezomib-induced phosphorylation of HSF1 on Ser326 contributes to multiple myeloma resistance to treatment (219). Silencing of HSF1 sensitized multiple myeloma cells to bortezomib treatment (219). Phosphorylation of HSF1 on Thr120 promotes breast cancer tumorigenesis compared to an overexpression of WT HSF1 (141). Another phosphorylation site on S419 was found at higher levels on melanoma cell lines, and the substitution of HSF1-Ser419 reduced the proliferation of these melanoma cell lines (138).

CsA, an immunosuppressant, enhances the phosphorylation of the negative activator site on HSF1, causing the death of HeLa cancer cells (137). Thus, CsA, *via* the inhibition of HSF1 through phosphorylation, could be used as chemotherapy to decrease tumor progression. HSF1 modulates the effects of cisplatin, an alkylating chemotherapy agent for oral squamous cell carcinoma, acting on PD-L1 (220). Additionally, cisplatin activates HSF1 by phosphorylating S326, leading to an upregulation of PD-L1 surface expression (220). Therefore, modulating HSF1 phosphorylation, especially of S326, is a promising chemotherapy target. One caveat to specifically targeting HSF1 PTMs is that some PTMs increase transcriptional activity while other PTM sites decrease activity, requiring specific PTM inhibitors to ensure a pro-cancer environment is not promoted with drug treatment (133). Because of HSF1’s role in promoting HSP70 and HSP90 refolding chaperone activity, inhibiting HSF1 would likely shift the preference of HSP70 and HSP90 towards CHIP and substrate degradation, thus providing a promising cancer treatment strategy.

### Heat shock protein 40

Targeting HSP40s would affect the refolding chaperone activity of HSP40 and ultimately decrease cancer cell proliferation. For example, inhibition of HSP40 results in a depletion of mutant p53 and reduced cancer cell proliferation in a panel of cancer cells (221). HSP40 controls the fate of the misfolded mutant p53, promoting the accumulation of this protein (222). Therefore, blocking the refolding activity of HSP40 would shift the chaperone preference towards CHIP and result in the degradation of cancer-promoting substrates. Inhibiting the interaction of the HSP70–HSP40 complex and its substrates decreased cancer stem cell growth, again highlighting how a shift towards CHIP and degradation may be necessary for cancer cell progression (223).

### Small HSPs

Specific drug targets are needed to ensure effective anticancer therapies because of the broad range of cellular roles for sHSPs. Selective inhibitors targeted towards other sHSPs have been challenging to create; thus, most research has focused on HSP27 as a target for drug therapies to reduce expression or inhibit action (224). Because of its role in cell death and PQC, HSP27 is a promising drug target for chemotherapies (163, 165). However, HSP27 lacks an ATP-binding domain; therefore, targeted chemotherapies likely need to focus on altering HSP27 function, such as targeting phosphorylation or dimerization (225). Small molecule inhibitors, peptides, and antisense oligonucleotides targeted to HSP27 mRNA have effectively decreased cancer proliferation. However, only the antisense oligonucleotide treatment is currently in clinical trials (226). Small molecule inhibitors such as quercetin bind directly to the sHSPs and inhibit their function. Quercetin works by inhibiting HSP27 phosphorylation and, therefore, its expression (227). It has also shown promising results in ovarian and breast cancer (228, 229). ***Peptides are designed to bind to specific protein domains, but little preclinical work has explored using peptide targets to sHSPs*** (224). sHSPs play a role in the formation, folding, and refolding of proteins. Therefore, chemically shifting the activity towards CHIP to promote degradation through sHSP inhibition would benefit cancer treatment.

## Conclusions

The HSR is modulated by various PTMs that affect protein interactions, outcomes on substrates, and disease progression. These proteins and their alterations are identified as potential targets for cancer therapy. A deeper understanding of this aspect of cellular PQC could lead to more effective pharmacological strategies. One area of further exploration is the HSP110 family of chaperones protein, which is not highlighted in this review but is still an essential system component. ***Although large HSPs were discovered in the 1990s, little research has investigated the PTMs on these proteins, highlighting a gap in knowledge in our understanding of the complete HSR system*** (230). HSP110 is the only protein in the HSP family with a mutation linked to the progression of colorectal cancer (231). Therefore, targeting this protein could be beneficial in treating colorectal cancer harboring this mutation. ***Research on large HSPs in cancer, specifically their role in cancer immunotherapy, demonstrates they are essential and influence cancer progression, emphasizing a key area of further study.***

Current research on the HSR often focuses on isolated components. However, this review underscores the complex interaction among all molecular entities involved and the importance of understanding the entire system for developing drug therapies. It is noted that encouraging the transition from HSP70/90 binding with HOP to CHIP could be advantageous in most cancer treatments. Nonetheless, modifying one element of the system may impact the overall response and significantly alter the therapeutic effects of drugs. Therefore, further research is necessary to grasp the dynamics between HSR components. Our review synthesizes critical elements of the HSR and how their alterations influence interactions, which could pave the way for novel therapeutic strategies against diseases like cancer. Future studies should consider the entire HSR network to fully comprehend the mechanisms of therapies that lead to successful treatment outcomes.

## Conflict of interests

The authors declare that they have no conflicts of interests with the contents of this article.
